# Serum a proliferation-inducing ligand and MicroRNA-223 are associated with rheumatoid arthritis: diagnostic and prognostic implications

**DOI:** 10.1186/s10020-020-00199-7

**Published:** 2020-10-01

**Authors:** Mohamed Taha, Olfat Gamil Shaker, Enas Abdelsalam, Noha Taha

**Affiliations:** 1 Biochemistry Department, Faculty of Pharmacy, Cairo University, 23 Kasr Al Ainy Street, Cairo, 11562 Egypt; 2 Medical Biochemistry and Molecular Biology Department, Faculty of Medicine, Cairo University, Cairo, Egypt; 3 National Institute of Diabetes and Endocrinology, Cairo, Egypt; 4 Internal Medicine Department, Faculty of Medicine, Cairo University, Cairo, Egypt

**Keywords:** APRIL, Autoimmune disease, Cytokine, miRNAs, Rheumatid

## Abstract

**Background:**

Current blood-based tests for rheumatoid arthritis (RA) have inherent limitations, necessitating the need for additional new biomarkers for its diagnosis and monitoring disease activity and responsiveness to therapy. MicroRNAs (miRNAs) and a proliferation-inducing ligand (APRIL) are deregulated in RA and were linked to its pathogenesis. This study investigated serum levels of APRIL, miR-223 and miR-155 in RA patients, their potential as diagnostic and prognostic biomarkers, and their correlation with disease activity and clinicopathological data.

**Methods:**

One hundred and twenty Egyptian patients with RA and 130 healthy controls were included. Serum miRNAs and APRIL were assayed by RT-qPCR and ELISA, respectively.

**Results:**

Serum APRIL and miR-223 were significantly upregulated, while miR-155 was unchanged in RA patients compared to controls. Serum miR-223 discriminated RA patients from controls with AUC = 0.85, whereas serum APRIL superiorly distinguished the two groups with AUC = 1 (sensitivity and specificity = 100% at cutoff> 4.19 ng/ml) by receiver-operating-characteristic analysis. Serum miR-223 was a significant predictor for RA diagnosis in multivariate logistic regression analysis. In RA group, serum APRIL was positively correlated with disease activity score (DAS28-CRP). Serum miR-223 expression was positively correlated with serum miR-155, APRIL levels and with the presence of subcutaneous nodules. Serum miR-155 levels were correlated with antinuclear antibody titer in reverse direction.

**Conclusion:**

Our results suggest serum APRIL and miR-223 could serve as potential biomarkers of RA, with miR-223 as a predictor of RA risk and APRIL as an excellent biomarker of disease activity. Our data could be implicated for accurate and blood-based non-invasive diagnosis and prognosis of RA.

## Introduction

Rheumatoid arthritis (RA) is a debilitating chronic inflammatory condition of the joints that affects approximately 1% of the world’s population (Ogrendik 2013) and about 0.3% of the Egyptian population (Gamal et al. 2016). It is a systemic autoimmune disease characterized by inflammation and hyperplasia, hypertrophy and angiogenesis of synovial tissues, with cartilage and bone destruction that lead to joint swelling, pain and stiffness (Ogrendik 2013; Gamal et al. 2016).

The genesis of RA involves complex multifactorial steps in which an interplay exists between genetic, epigenetic, immunological and environmental factors, but knowledge of the full molecular basis of RA is still incomplete (Senousy et al. 2019). In addition, the current diagnostic methods of RA are unreliable and lack sufficient sensitivity and specificity. Blood tests include erythrocyte sedimentation rate (ESR) and C-reactive protein (CRP), which are non-specific markers of systemic inflammation*.* In addition*,* serum levels of autoantibodies, rheumatoid factor (RF) and anti-cyclic citrullinated peptide antibodies (anti-CCP) were also developed as early biomarkers for diagnosis of RA (Bukhari et al. 2002). However, some patients have RA without having anti-CCP or RF (Kawano et al. 2007). Thereby, new diagnostic markers for RA are urgently needed. Elucidating the molecular mechanisms underlying RA development and progression may unravel new diagnostic and prognostic biomarkers or therapeutic targets for RA.

MicroRNAs (miRNAs) are endogenous small ~ 22 nucleotide non-coding RNAs that fine tune gene expression by degrading or suppressing the translation of target mRNAs. miRNAs can control many immune processes, including T- and B-cell development and maturation, antigen presentation, Toll-like receptor signaling and pro-inflammatory cytokine production, immunoglobulin class-switch recombination in B-cells, and T-cell receptor signaling (Ceribelli et al. 2012). Differential expression of non-coding RNAs, including miRNAs were found in patients affected by several autoimmune diseases, and were linked to the pathogenesis of these conditions (Senousy et al. 2019; Senousy et al. 2020; Abd-Elmawla et al. 2020). Indeed, dysregulated miRNA expression has been shown to be implicated into the molecular mechanisms of RA (Tavasolian et al. 2018). Noteworthy, specific miRNAs such as miR-146a and miR-155 appear to be systematically dysregulated in RA (Zhou et al. 2014). Furthermore, circulating miRNAs are deregulated in RA and are emerging as promising stable and easily detectable blood-based non-invasive biomarkers for RA diagnosis, prognosis and response to therapy (Tavasolian et al. 2018; Murata et al. 2010; Filková et al. 2014). Thereby, profiling of circulating RA-related miRNAs may identify new molecular biomarkers for RA as well as future targets for new therapeutic approaches.

Although the precise mechanisms leading to RA remain incompletely understood, extensive evidence suggests that B-cells play an important role in its pathogenesis. miR-223 is a hematopoietic miRNA that is important for B-cell differentiation and development (Johnnidis et al. 2008; Sun et al. 2010). In addition, the B cell-stimulating molecules, BAFF (B-cell activating factor) and a proliferation-inducing ligand (APRIL) are critical factors in the maintenance of the B cell pool and humoral immunity. APRIL is a member of the tumor necrosis factor (TNF) family that regulates B-cell maturation, survival, and function. APRIL is expressed by myeloid cells, notably neutrophils, T-cells, dendritic cells, monocytes, and macrophages but not by B-cells (Slifka et al. 1998). APRIL become an active legend as homotrimers in the circulation where it is recognized by B-cell maturation antigen (BCMA) and transmembrane activator and calcium-modulating cyclophilin ligand interactor (TACI) on B-cell surface to promote B-cells differentiation and proliferation (Seshasayee et al. 2003). APRIL also serves as potent co-activator to augment immunoglobulin production (Seshasayee et al. 2003). APRIL can maintain the activation of B cells, thus enhancing autoimmune diseases (Hofmann et al. 2018). Indeed, elevated levels of APRIL were detected in the sera of patients with RA, systemic lupus erythematosus (SLE), IgA nephropathy and Sjögren’s syndrome (Zhao et al. 2014; Treamtrakanpon et al. 2012; Samy et al. 2017). As such, this molecule along with BAFF were rational targets for new therapies in B cell-driven autoimmune diseases, such as the BAFF/APRIL dual inhibitor, atacicept and the BAFF inhibitor, belimumab which is approved as an add-on therapy for active SLE (Samy et al. 2017), however, more preclinical and clinical studies on APRIL are still needed in RA.

Thus, this study aimed to evaluate serum levels of APRIL as well as selected miRNAs; miR-155 and miR-223 in Egyptian patients with RA, their potential as diagnostic and prognostic biomarkers, and their correlation with disease activity and clinicopathological parameters in RA patients.

## Materials and methods

### Patients

This cross-sectional study included 120 patients with RA (90% women vs 10% men, age 19–60 years) in addition to 130 age and gender-matched healthy volunteers (83% women vs 17% men, age 20–59 years). RA patients were recruited from the rheumatology unit of Kasr Al-Ainy hospital, Cairo, Egypt and fulfilled the American College of Rheumatology (ACR 1987) criteria for RA. Full history taking and clinical examination were performed to the RA patients. All patients were receiving disease-modifying anti-rheumatic drugs. Disease activity was assessed by measuring the disease activity score for 28 joints (DAS28) by C-reactive protein (CRP) (Prevoo et al. 1995). The DAS28 considers 28 tender and swollen joint counts, general health; patient assessment of disease activity using the 100 mm visual analog scale (VAS) with 0 = best, 100 = worst, plus levels of an acute phase reactant (CRP [mg/l]).

By questionnaire, we confirmed that the volunteers had no history of immunological diseases or were not being treated for arthralgia, heart failure, renal failure, or autoimmune disease and were free from other inflammatory conditions.

### Laboratory investigations

Patients were subjected to routine laboratory investigations, including complete blood count (CBC), erythrocyte sedimentation rate (ESR) (mm/1st hour, Westergren method), serum CRP, RF, antinuclear antibody (ANA), alanine aminotransferase (ALT) activity, and creatinine levels.

### Serum APRIL assay

Measurement of serum APRIL was done for both patients and healthy controls by ELISA technique according to the manufacturer’s instructions (Thermofisher, USA).

### Serum miRNAs assay

#### RNA extraction and reverse transcription

Total RNA was extracted from serum by miRNeasy extraction kit (Qiagen, Valenica, CA) using QIAzol lysis reagent according to the manufacturer’s instructions. RNA concentration and purity were determined using NanoDrop2000 (Thermo scientific, USA). Reverse transcription (RT) was carried out on 100 ng of total RNA per 20 μl RT reaction volume (incubated for 60 min at 37 °C and 5 min at 95 °C) using miScript II RT Kit (Qiagen, Valenica, CA) according to the manufacturer’s instructions.

#### Real-time PCR

Serum expression levels of mature miRNAs, miR-223 and miR-155 were evaluated using miScript miRNA PCR primer assays and miScript SYBER green PCR kit (Qiagen, Valenica, CA) according to the manufacturer’s protocol. The housekeeping miRNA SNORD68 was used as the internal control as previously described (Shaker and Senousy 2017). Real-time PCR was performed using Rotor gene Q System (Qiagen, Valenica, CA) using the following conditions: 95 °C for 30 min, followed by 40 cycles at 94 °C for 15 s, 55 °C for 30 s, and 70 °C for 30 s. The cycle threshold (Ct) is the number of cycles required for the fluorescent signal to cross the threshold in real-time PCR. ∆Ct was calculated by subtracting the Ct values of miRNA SNORD68 from the Ct values of the target miRNAs. Fold change was calculated using 2^-∆∆Ct^ for relative quantification.

### Statistical analysis

Statistical analyses were performed using computer program Statistical Package for the Social Science (SPSS, Chicago, IL) software version-15 for Microsoft Windows and GraphPad Prism-5.0 (GraphPad Software, CA). Values were expressed as mean ± standard deviation (SD), median interquartile range [median (25–75% percentiles)] or number (percentage) when appropriate. Categorical data were compared by Fischer exact test. Shapiro-Wilk and Kolmogorov-Smirnov tests were performed to test data normality. Clinical data were normally distributed and compared using Student’s t test or one-way analysis of variance when appropriate. The miRNA data were not normally distributed, so miRNA levels from independent samples were compared using the non-parametric Mann-Whitney *U*-test or Kruskal-Wallis test when appropriate. The diagnostic accuracy of miRNAs was evaluated by receiver-operating-characteristic (ROC) analysis and the area under the curve (AUC) was calculated. AUC < 0.6 was considered non-significant, 0.7–0.9 was considered as a potential discriminator, and > 0.9 was considered as an excellent discriminator. Comparison of AUCs was done using the non-parametric method of Delong. Univariate and multivariate logistic regression analyses were done to identify predictor variables associated with the risk of RA using age and sex as covariates. Data that were significant in the univariate analysis were then entered multivariate analysis to select the best model containing the final independent variables. Correlations between parameters were determined by Spearman correlation. *P* < 0.05 was considered significant, with a 95% confidence interval (CI).

## Results

### Demographic, clinical and pathological characteristics of RA patients

The demographic and clinical data of RA patients and healthy controls are shown in Table 1. 60% of RA patients were having considerable disease activity (DAS28-CRP score ≥ 2.3; low, mild, or high activity), with 27.5% were having high disease activity (DAS28-CRP > 4.1). Regarding clinicopathological data, 75, 70, 25, 20% of RA patients were having arthritis, deformities, subcutaneous nodules, and extra-articular manifestations, respectively (Table 1).

**Table 1:** Characteristics of RA patients and healthy controls

Parameter	RA patients (*n* = 120)	Healthy controls (*n* = 130)	*P* value
Sex			0.13
Male, n (%)	12 (10%)	22 (17%)	
Female, n (%)	108 (90%)	108 (83%)	
Age (years)	38.25 ± 10.13	39.9 ± 15.2	0.32
Range	(19–60)	(20–59)	
Hemoglobin (g/dl)	11.83 ± 1.63	12.3 ± 2.5	0.08
Total leukocyte count (× 10^3^/mm^3^)	7.72 ± 2.48	8 ± 3.1	0.43
Platelet count (×10^3^/mm^3^)	279.6 ± 80.85	288.26 ± 75.2	0.4
ALT (U/l)	25.63 ± 10.89	27.51 ± 11.48	0.19
Creatinine (mg/dl)	0.82 ± 0.22	0.9 ± 0.42	0.06
Disease duration (y)	6.22 ± 4.45	NA	
MS (min)	25.38 ± 25.18	NA	
ESR (mm)	39.13 ± 23.6	NA	
RF, n (%)		NA	
Positive	90 (75)		
Negative	30 (25)		
ANA, n (%)		NA	
Positive	18 (15)		
Negative	102 (85)		
DAS28-CRP	2.98 ± 1.73	NA	
Remission < 2.3	48 (40)		
Low disease activity 2.3–2.7	9 (7.5)		
Mild disease activity 2.7–4.1	30 (25)		
High disease activity > 4.1	33 (27.5)		
VAS (mm)	5.22 ± 3.34	NA	
Swollen joint count (SJC)	5.33 ± 5.66	NA	
Tender joint count (TJC)	5.17 ± 5.45	NA	
Arthritis, n (%)		NA	
Severe	87 (72.5)		
Mild	33 (27.5)		
Deformities, n (%)		NA	
Yes	84 (70)		
No	36 (30)		
Fever, n (%)			
Yes	15 (12.5)		
No	105 (87.5)	NA	
Subcutaneous nodules, n (%)			
Yes	30 (25)		
No	90 (75)		
Extra articular manifestations, n (%)		NA	
Yes	24 (20)		
No	96 (80)		
Treatment	NA		
Steroids only	6 (5)		
MTX only	60 (50)		
HQN only	6 (5)		
MTX + HQN	24 (20)		
MTX + steroids	24 (20)		

Data are expressed by mean ± SD or number (percentage). *ALT* alanine aminotransferase, *ANA* antinuclear antibody, *CRP* C-reactive protein, *DAS28* 28-Joint disease activity score, *ESR* erythrocyte sedimentation rate, *HQN* hydroquinone, *MS* morning stiffness, *MTX* methotrexate, *NA* not applicable, *RF* rheumatoid factor, *VAS* visual analogue scale for general health

### Serum miRNAs levels

The present study has demonstrated the expression profile of two miRNAs, miR-223 and miR-155. Serum miR-223 expression levels were significantly upregulated in RA patients compared to healthy controls with median fold change of 22 (*P* < 0.0001). miR-155 level was not significantly different between RA patients and healthy controls (*P* = 0.15) (Table 2).

**Table 2:** Serum miRNA levels in RA patients

Fold change of miRNAs in RA compared to healthy controls			
miRNA	Fold change	Fold regulation	*P* value
	Median (25–75% percentiles)		
miR-223	22 (7.08–66.4)	22	< 0.0001*
miR-155	1.5 (0.41–7.92)	1.5	0.15

Data are expressed as median (25–75% percentiles) and were analyzed by Mann-Whitney *U*-test. RA group = 120, healthy controls, *n* = 130. * indicates statistical significance (*P* < 0.05)

### Serum APRIL level

**Fig. 1:**
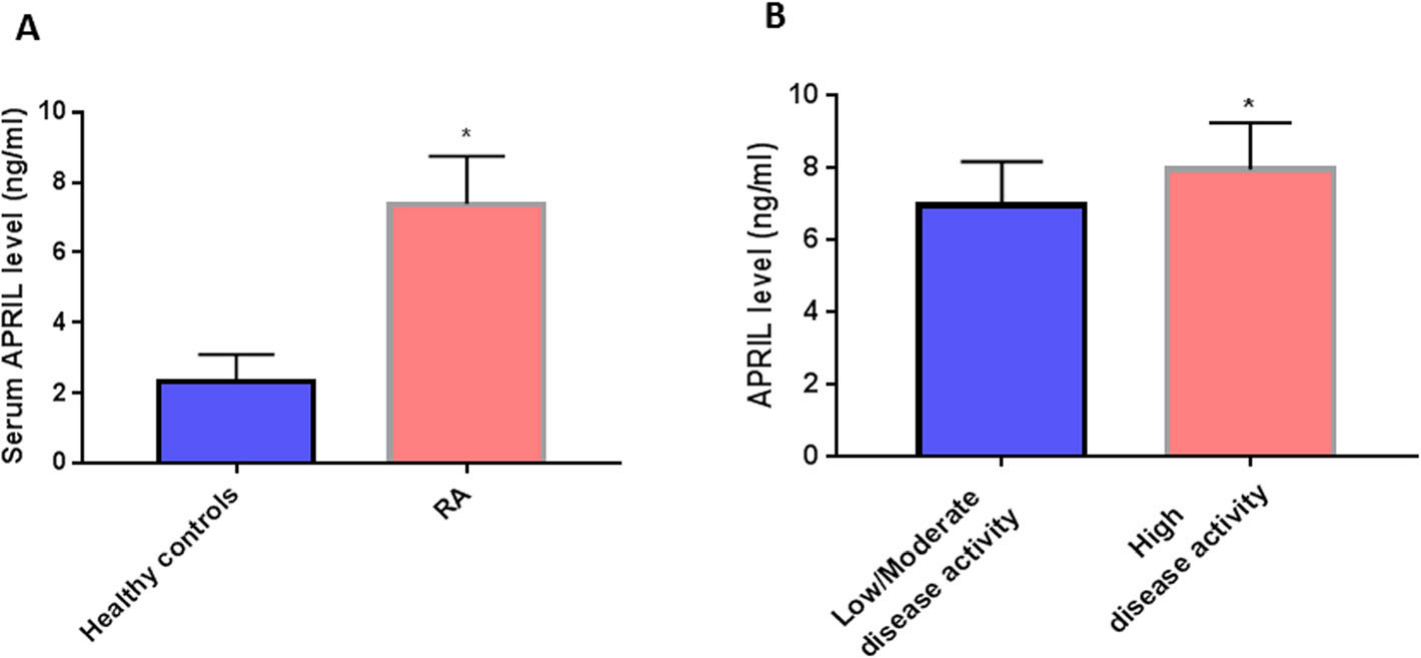
Serum APRIL level in RA patients and its relation to disease activity. **a** Serum APRIL in RA (*n* = 120) compared to healthy controls (*n* = 130), *P* < 0.0001. **b** Serum APRIL in low/mild disease activity patients (DAS28-CRP = 2.3–4.1, *n* = 39) vs high disease activity patients (DAS28-CRP > 4.1, *n* = 33), *P* = 0.0012. Data are expressed as mean ± SD and were analyzed by Student’s t test. * indicates statistical significance, *P* < 0.05

Serum APRIL was significantly upregulated in RA patients compared to healthy controls (*P* < 0.0001) with mean ± SD = 7.39 ± 1.2 vs 2.3 ± 0.069 ng/ml, respectively (Fig. 1a).

Furthermore, the potential influence of medical therapies on APRIL, and miR-223 and miR-155 expression levels was evaluated. However, there were no significant differences in their levels between patients receiving different treatments at the time of blood collection (*P* > 0.05). (Supplementary Figure S1).

### Correlations between studied serum miRNAs and APRIL with clinicopathological data

We examined significant correlations between investigated parameters in RA patients with considerable disease activity (DAS28-CRP ≥ 2.3) (Table 3). We found that serum APRIL was positively correlated with disease activity score (DAS28-CRP) (*r* = 0.27, *P* = 0.02), with APLIL levels mean ± SD = 7.97 ± 1.29 vs 6.97 ± 1.2, *P* = 0.0012 in high vs low/mild disease activity patients, respectively (Fig. 1b). Serum miR-223 was positively correlated with miR-155 (*r* = 0.391, *P* = 0.003) and APRIL levels (*r* = 0.29, *P* = 0.038). miR-223 was also positively correlated with the presence of subcutaneous nodules (*r* = 0.39, *P* = 0.02). Serum miR-155 levels were correlated with ANA titer in reverse direction (*r* = − 0.43, *P* = 0.005). There were no significant correlations for serum miRNAs with disease activity.

**Table 3:** Correlation of serum miRNAs, APRIL and clinicopathological data in RA patients

		APRIL	miR-223	miR-155
APRIL	r		0.29	−0.05
	P		0.038*	0.76
miR-223	r	0.29		0.391
	P	0.038*		0.003*
miR-155	r	−0.05	0.391	
	P	0.76	0.003*	
ESR	r	0.17	0.023	0.03
	P	0.27	0.88	0.87
RF	r	0.002	−0.18	0.19
	P	0.98	0.26	0.22
ANA	r	−0.04	− 0.19	− 0.43
	P	0.79	0.23	0.005*
VAS	r	0.23	0.05	−0.1
	P	0.06	0.76	0.55
DAS28-CRP	r	0.27	0.045	−0.02
	P	0.02*	0.78	0.89
TJC	r	0.2	0.03	0.05
	P	0.16	0.85	0.75
SJC	r	0.18	0.15	0.053
	P	0.25	0.33	0.74
Disease duration	r	−0.07	−0.12	0.02
	P	0.65	0.42	0.90
MS by min	r	0.12	0.07	0.05
	P	0.42	0.63	0.75
Arthritis	r	0.05	−0.14	0.03
	P	0.75	0.37	0.85
Deformities	r	0.08	0.04	−0.05
	P	0.6	0.82	0.77
Fever	r	0.23	0.23	−0.12
	P	0.17	0.17	0.44
Subcutaneous nodules	r	0.005	0.39	0.21
	P	0.97	0.02*	0.19
Extra articular	r	0.19	0.12	−0.03
manifestations	P	0.22	0.44	0.86

r = spearman rho coefficient, correlations were done using Spearman correlation

*indicates statistical significance, *P* < 0.05. Correlations were done in RA patients with considerable disease activity (DAS-28-CRP ≥ 2.3), *n* = 72. *ANA* antinuclear antibody, *DAS28* 28-Joint disease activity score, *ESR* erythrocyte sedimentation rate, *MS* morning stiffness, *RF* rheumatoid factor, *SJC* swollen joint count, *TJC* tender joint count, *VAS* visual analogue scale for general health. Categorical data was considered zero (for absent) or 1 (for present) in the correlation

### Results of ROC curve analysis for serum miRNAs and APRIL

**Fig. 2:**
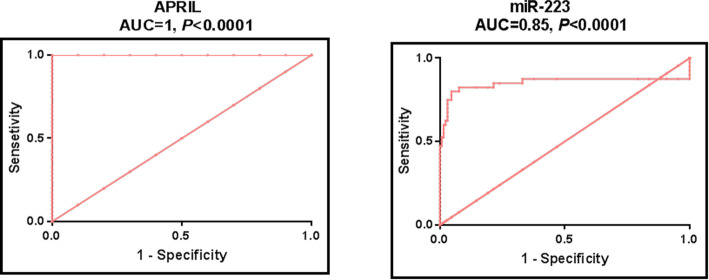
Serum miR-223 and APRIL levels as biomarkers of RA. ROC curve analysis of serum miR-223 and APRIL levels to discriminate RA patients (*n* = 120) from healthy controls (*n* = 130)

Our study further evaluated studied serum miRNAs as well as APRIL levels as potential biomarkers of RA using ROC analysis (Fig. 2). Results revealed that miR-223 discriminated RA patients from controls with AUC = 0.85, *P* < 0.0001, suggesting it as a potential discriminator. The calculated sensitivities, specificities, positive predictive values (certainty to prove RA) and negative predictive values (certainty to exclude RA) were 80, 95.38, 94.11, and 83.78%, respectively at cutoff value of > 2.5 fold (Table 4).

**Table 4:** Diagnostic performances of serum miR-223 and APRIL to discriminate RA patients from healthy controls

miRNA	AUC (95%CI)	*P* value	Best cutoff value	Sensitivity %	Specificity %	PPV %	NPV %
miR-223	0.85 (0.79 to 0.91)	< 0.0001	> 2.5 fold	80	95.38	94.11	83.78
APRIL	1* (1 to 1)	< 0.0001	> 4.19 ng/ml	100	100	100	100

*PPV* Positive predictive value, *NPV* negative predictive value. *significant difference between AUCs, *P* < 0.0001. RA, *n* = 120; healthy controls, *n* = 130

Interestingly, our results showed that serum APRIL discriminated RA patients from controls with AUC = 1, with sensitivity, specificity, positive and negative predictive values of 100% at cutoff value of > 4.19 ng/ml (Table 4), suggesting APRIL as an excellent discriminator.

Comparison of AUCs revealed that serum APRIL demonstrated significantly higher diagnostic accuracy than miR-223 (difference = 0.15, *P* < 0.0001) to distinguish RA patients from healthy control (Table 4).

### Results of logistic regression analysis

Furthermore, we conducted logistic regression analysis to determine the predictor variables for the risk of being diagnosed with RA using age and sex as confounders (Table 5). Results revealed that that serum miR-223 and APRIL were selected as significant predictor variables for RA diagnosis in the univariate analysis. In the multivariate analysis, only miR-233 was turned out to be an independent positive predictor of RA diagnosis.

**Table 5:** Logistic regression analysis

Parameter	Coefficient	SE	*P*^a^ value	Odds ratio	Odds ratio (95% CI)
**Univariate analysis**					
miR-223	0.1	0.023	0.005*	1.35	1.1–1.9
APRIL	0.46	0.24	0.04*	1.52	1.01–2.25
**Multivariate analysis**					
miR-223	0.12	0.032	0.008*	1.4	1.13–1.99
APRIL	0.58	0.38	0.09	1.6	0.9–3.17
Constant	2.1				

^a^ adjusted for age and sex. * indicates statistical significance, *P* < 0.05. Logistic regression analysis was done in RA group, *n* = 120 vs healthy controls, *n* = 130, including age and sex as confounders. -2 Log Likelihood ratio, *P* < 0.0001

## Discussion

The current biomarkers for RA have inherent limitations. Consequently, there is a need for additional new biomarkers for diagnosis and monitoring disease activity and responsiveness to therapy of RA patients.

The present study revealed that miR-223 was differentially expressed in sera of RA patients and was a positive predictor of RA diagnosis in multivariate logistic regression analysis, implicating miR-223 as potential biomarker of RA. Furthermore, our study revealed differential expression of serum APRIL in RA patients, suggesting APRIL as surrogate biomarker for RA. Indeed, serum APRIL performed very high in RA diagnosis (AUC = 1, sensitivity and specificity = 100%), and was better than miR-223 (AUC = 0.85), implicating APRIL as excellent new blood-based marker for RA diagnosis. Interestingly, APRIL was correlated with disease activity, indicating that APRIL could serve as a biomarker of RA prognosis. These results enroll APRIL and miR-223 as possible therapeutic targets for RA treatment. Perhaps combination of these markers as well as their addition to other serological markers may improve the diagnostic accuracy for RA detection, however this needs further investigation.

Our study demonstrated an upregulation of serum APRIL in RA patients that was also positively correlated with disease activity (DAS28-CRP). Our results were concordant with earlier reports demonstrating high serum APRIL levels in serum and synovial tissue of RA patients (Tayel et al. 2013; Boghdadi et al. 2015). Our results could be explained on the basis that in the early phase of RA disease, the APRIL is produced significantly by dendritic cells, which leads to an increase in the proliferation of B cells; and ultimately, B cells are differentiated by APRIL producing autoantibodies (Shabgah et al. 2019). Macrophages, on the other hand, are considered to be the source of APRIL in the confirmed phase of the disease (Ancuta et al. 2017), causes the accumulation of plasma cells in the joint, further increasing the production of inflammatory cytokines such as TNF, IL-1, and IL-6 (Zhao et al. 2014).

In addition, APRIL promotes proliferation, secretion and invasion of fibroblast-like synoviocytes in adjuvant-induced arthritis (Chang et al. 2015). Previous studies also demonstrated that serum APRIL levels were higher in seropositive RA patients than those of seronegative RA patients, suggesting that APRIL may participate in the formation of seropositive RA (Tayel et al. 2013; Boghdadi et al. 2015). Similar to our results, serum APRIL was also correlated positively with disease activity; SJC, VAS and simplified disease activity index (Boghdadi et al. 2015), while synovial APRIL was correlated with ESR, CRP, anti-CCP and DAS28 (Tayel et al. 2013). Together, these results suggest APRIL as a good marker for joint injury. Additionally, we are the first to demonstrate the high diagnostic performance of APRIL in RA that performed better than miRNAs. This could be attributed to the heterogeneity of miRNA data in studied population and the use of normalizing internal control.

We recorded an upregulation of serum miRNA-223 in RA patients that was correlated with the presence of subcutaneous nodules, linking this miRNA to RA development and severity. Our results are in agreement with previous studies demonstrating overexpression of miR-223 in blood and synovial T-lymphocytes from RA patients (Muscari et al. 2013; Fulci et al. 2010). Indeed, miR-223 was upregulated in serum and synovial CD3+ T lymphocytes from RA patients compared to healthy controls (Muscari et al. 2013). Furthermore, miR-223 was overexpressed in peripheral blood CD3+ and CD4+ naive T-lymphocytes of RA patients and contributed to the pathogenesis of the disease (Fulci et al. 2010). In addition, miR-223 was also intensely expressed in RA synovium; its overexpression in RA synovial fibroblasts suppressed osteoclastogenesis in vitro (Shibuya et al. 2013).

Our results are consistent with the role of miR-223 in RA pathogenesis. In fact, miR-223 is a hematopoietic miRNA that regulates progenitor cell proliferation, granulocyte function, macrophage differentiation and survival (Johnnidis et al. 2008; Ismail et al. 2013). miR-223 regulates inflammation and immunity by targeting different targets, including cytoplasmic activation/proliferation-associated protein-1 (Caprin-1), heat shock protein 90 (Hsp90), signal transducer and activator of transcription 5 (STAT5), CCAAT enhancer-binding protein-β (CEBP-β), nuclear factor I (NFI-A), and other transcription factors (Aziz 2016). miR-223 was also involved in limiting NOD-like receptor, pyrin domain containing 3 (NLRP3) inflammasome and pro-interleukine-1β cytokine production (Haneklaus et al. 2012). These data partially explain the observed positive correlation between miR-223 and the presence of nodules in our study. Notably, the most prominent cells in the nodule belong to the monocyte/macrophage lineage (Highton et al. 2007). It seems probable that miR-223 is expressed in the superficial and the sublining layers macrophages/monocytes, where it regulates macrophage differentiation.

Interestingly, we demonstrated a correlation between serum miR-223 and APRIL, suggesting that these molecules might synchronize to regulate B-cell activation, an important etiology of RA. Notably, miR-223 has important role in the B-cell development through activation of its targets, LIM domain only 2 (LMO2) and CEBP-β (Sun et al. 2010).

The present study demonstrated that serum miR-155 was not significantly different between RA patients and controls. Conversely, miR-155 was upregulated in serum and peripheral blood mononuclear cells (PBMCs) from RA patients compared to healthy controls (Pauley et al. 2008; Churov et al. 2015). The observed negative correlation between miR-155 and ANA titer could be explained on the basis that higher expression of miR-155 was reported in synovial cells and tissues, and was associated with the repression of matrix metalloproteinases production, thus leading to modulation of joint inflammation and consequently less severe disease (Kurowska-Stolarska et al. 2011). In fact, miR-155 is a proinflammatory miRNA that targets multiple genes, including regulatory proteins for myelopoiesis, leukemogenesis and inflammation (Katschke et al. 2001).

The current study also revealed that the tested miRNAs were positively correlated in RA, suggesting their concomitant dysregulation in RA. Indeed, both miR-223 and miR-155 were mechanistically linked to lymphocyte differentiation and activation at different levels (Johnnidis et al. 2008; Fulci et al. 2010; Rodriguez et al. 2007). However, neither of studied miRNAs was correlated with disease activity. Similarly, plasma miR-223 was not correlated to disease activity or inflammatory markers in RA patients (Andonian et al. 2019). In contrast, circulating miR-223 level in treatment naïve early RA patients was correlated with CRP and DAS28, and was considered a marker of disease activity and a possible predictor for disease outcome in early RA (Filková et al. 2014). This discrepancy could be due to different study populations, selection bias, disease duration; early or late disease, and whether recruited patients receive treatment.

Few studies have evaluated circulating miRNAs as diagnostic biomarkers of RA (Murata et al. 2010; Filková et al. 2014; Churov et al. 2015; Anaparti et al. 2017), but neither study evaluated the diagnostic performance of serum APRIL in RA and its correlation with serum miRNAs. Herein, we demonstrated serum APRIL as both a reliable biomarker for RA diagnosis and a marker of disease activity. In addition, our study is unique as we demonstrated a correlation between epigenetics and APRIL that may add complexity to the role of B-cell activation on the pathogenesis of RA.

Our study is limited by being a hospital-based study, selection bias has ineluctably occurred. Additional large-scale population studies are required to validate our findings. Future studies should also take into account the genetics of APRIL or its pathway as subsets of RA patients may benefit from specific APRIL blockade. Nevertheless, we believe that our findings are potentially sound for clinical and personalized medicine and have potential implications in diagnosis and prognosis of RA. Our findings may also support the new specific therapeutic strategies for RA through targeting APRIL or its pathway.

## Conclusion

Our results suggest serum APRIL and miR-223 could serve as potential biomarkers of RA, with miR-223 as a predictor of RA risk and APRIL as an excellent biomarker of disease activity. Our data could be implicated for accurate and blood-based non-invasive diagnosis and prognosis of RA.

## Supplementary information

**Additional file 1: Figure S1.** Effect of different treatment regimens on serum APRIL and studied miRNAs.

## Data Availability

All data generated or analysed during this study are included in this published article.

## Abbreviations

APRIL: A proliferation-inducing ligand
Anti-CCP: Anti-cyclic citrullinated peptide antibodies
CRP: C-reactive protein
ANA: Anti-nuclear antibody
DAS-28: Disease activity score for 28 joints
ESR: Erythrocyte sedimentation rate
miRNAs: microRNAs
RA: Rheumatoid arthritis
RF: Rheumatoid factor
TACI: Transmembrane activator and calcium-modulating cyclophilin ligand interactor
TLR: Toll-like receptor
