# Supplementary material for: Serum a proliferation-inducing ligand and MicroRNA-223 are associated with rheumatoid arthritis: diagnostic and prognostic implications
Source: Mol Med. 2020 Oct 1;26:92. doi: 10.1186/s10020-020-00199-7 (PMC7528601; doi:10.1186/s10020-020-00199-7)
Supplement: Supplementary file 1 — Additional file 1: Figure S1. Effect of different treatment regimens on serum APRIL and studied miRNAs. [file 10020_2020_199_MOESM1_ESM.docx]

**Serum a proliferation-inducing ligand and microRNA-223 are associated with rheumatoid arthritis: Diagnostic and prognostic implications**

Mohamed Taha^1*^, Olfat Gamil Shaker^2^, Enas Taha^3^, Noha Taha^4^

^1^Biochemistry Department, Faculty of Pharmacy, Cairo University, Cairo, Egypt

^2^Medical Biochemistry and Molecular Biology Department, Faculty of Medicine, Cairo University, Cairo, Egypt

^3^National Institute of Diabetes and Endocrinology, Cairo, Egypt

^4^Internal medicine department, Faculty of Medicine, Cairo University, Cairo, Egypt


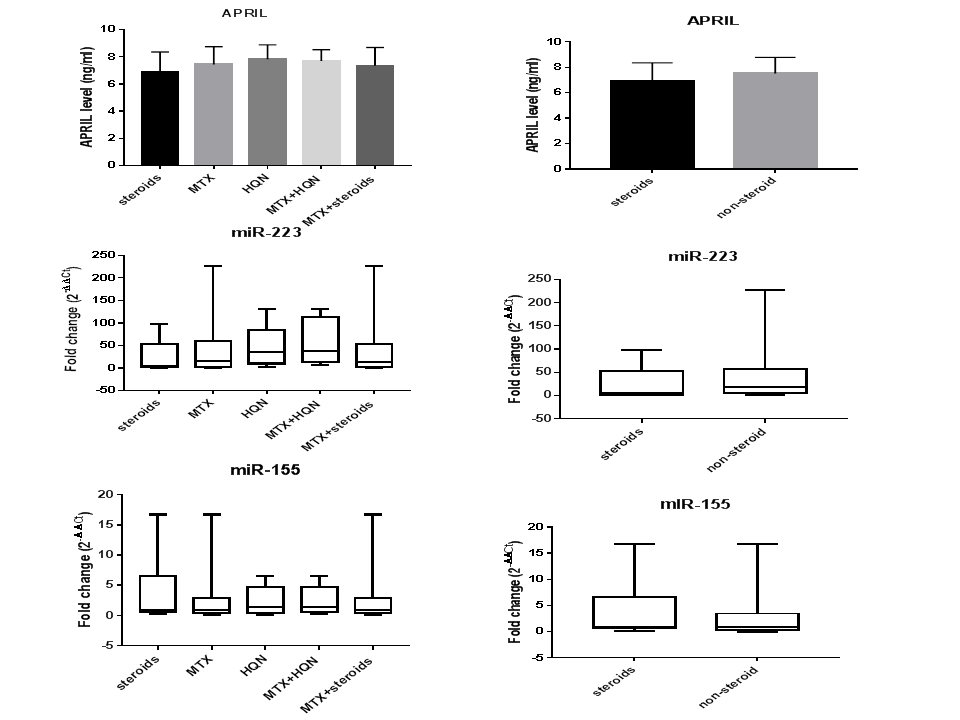


**Supplementary Figure S1. Effect of different treatment regimens on serum APRIL and studied miRNAs.** The data included any patient receiving steroid, n=30; methotrexate (MTX)=108; or hydroquinone (HQN), n=30; MTX+HQN, n=24; or MTX+steroids, n=24. APRIL data were expressed as mean±SD and were compared with ANOVA. miRNA data were expressed as box plots and were compared using kruskal-Wallis test.
